# Strong plasmon-exciton coupling in a hybrid system of gold nanostars and J-aggregates

**DOI:** 10.1186/1556-276X-8-134

**Published:** 2013-03-22

**Authors:** Dzmitry Melnikau, Diana Savateeva, Andrey Susha, Andrey L Rogach, Yury P Rakovich

**Affiliations:** 1 CIC nanoGune Consolider, Tolosa Hiribidea 76, Donostia-San Sebastian 20018, Spain; 2 Centro de Física de Materiales (MPC, CSIC-UPV/EHU), Donostia International Physics Center (DIPC), Po Manuel de Lardizabal 5, Donostia-San Sebastian 20018, Spain; 3 Centre for Functional Photonics (CFP), Department of Physics and Materials Science, City University of Hong Kong, Hong Kong, SAR, People's Republic of China; 4 IKERBASQUE, Basque Foundation for Science, Bilbao 48011, Spain

**Keywords:** Gold, Nanostars, Organic compounds, Plasmons, Rabi splitting, Fano effect

## Abstract

Hybrid materials formed by plasmonic nanostructures and J-aggregates provide a unique combination of highly localized and enhanced electromagnetic field in metal constituent with large oscillator strength and extremely narrow exciton band of the organic component. The coherent coupling of localized plasmons of the multispiked gold nanoparticles (nanostars) and excitons of JC1 dye J-aggregates results in a Rabi splitting reaching 260 meV. Importantly, broad absorption features of nanostars extending over a visible and near-infrared spectral range allowed us to demonstrate double Rabi splitting resulting from the simultaneous coherent coupling between plasmons of the nanostars and excitons of J-aggregates of two different cyanine dyes.

## Background

**Figure 1:**
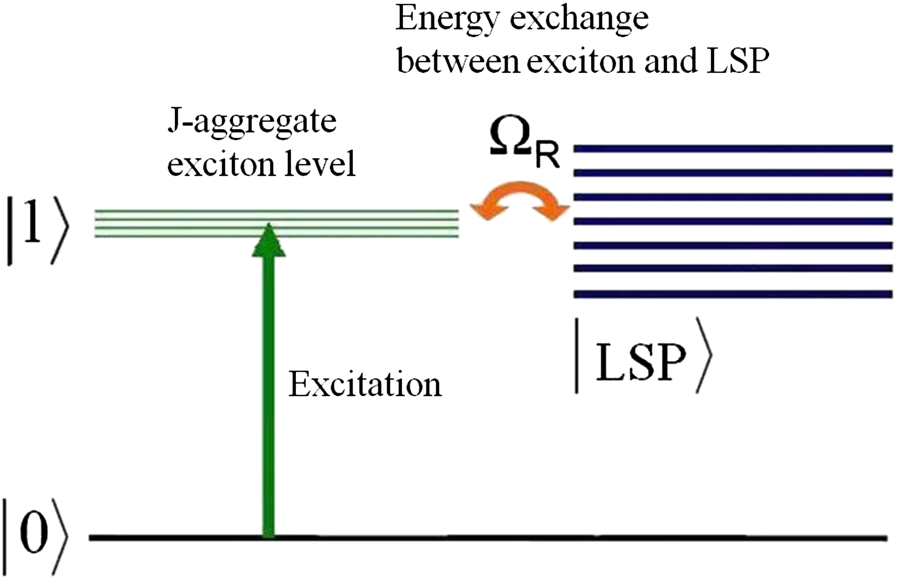
Schematic of the optical transitions in metal/J-aggregate hybrid nanostructure.

J-aggregates of the organic dyes are of significant interest for the development of advanced photonic technologies, thanks to their ability to delocalize and migrate excitonic energy over a large number of aggregated dye molecules [1]. The hybridization of electronic states in strongly coupled hybrid nanosystems consisting of plasmonic nanostructures and J-aggregates results in intriguing quantum electrodynamics phenomena such as Rabi splitting [2]. Optical transitions in this type of hybrid system are schematically illustrated in Figure 1. The absorption spectrum of J-aggregates is governed by optical transition from the electronic ground state │0〉 to a band of localized exciton states │1〉 , which is inhomogeneously broadened due to some energetic disorder which affects exciton localization [3]. In a hybrid metal/J-aggregate system, these exciton excitations can be strongly coupled to the localized surface plasmon (LSP) excitations of a metal nanostructure with a coherent exchange of energy between the excitonic and plasmonic systems, the so-called Rabi oscillation with frequency Ω_R_. This periodic energy exchange has an analogy with two coupled oscillators where new eigenmodes of the system arise, manifesting itself in the appearance of a double-peaked feature in transmission or absorption spectra [2]. The strength of the coupling is characterized by the value of energy of Rabi splitting, which can be estimated from the spectral distance between these two peaks.

In the strong coupling regime, the value of Rabi splitting depends on the oscillator strength of the exciton as well as on the increase in the local density of the electromagnetic modes and field enhancement both provided by noble metal nanostructures. To date, Rabi splitting arising from coherent coupling between electronic polarizations of plasmonic systems and molecular excitons in J-aggregates of cyanine dyes has been demonstrated for a variety of metal constituents, such as Au, Ag, and Au/Ag colloidal nanoparticles [4,5], core-shell Au and Ag nanoparticles [6,7], Ag films [8], spherical nanovoids in Au films [9], Au nanoshells [10], Au nanorods [11,12], and arrays of Ag nanodisks [13].

Among different plasmonic nanostructures, multispiked gold nanoparticles with a star-like shape [14-17] are of particular interest for the development of photonic devices and sensors based on the strong coupling phenomenon. These nanoparticles consist of a core with typically five to eight arms [18], whose sharp tips give rise to the strong spatial confinement of the electromagnetic field, with enhancement factors similar to those in metallic nanoshell dimers [19,20]. The coexistence of different plasmon resonances resulting from the hybridization of the core and the individual tips results in the increased number of localized plasmonic modes [19,21] (as compared to spherical nanoparticles or nanorods) available for the coherent interaction with quantum emitters. Moreover, the hybridization of plasmons localized at the core and the tips of the stars results in the increased effective dipole moment of the tip plasmons and the enlarged cross section for plasmon excitation [19]. In this study, we use these advantages of gold nanostars to develop their hybrid structures with J-aggregates of different organic dyes operating in the strong coupling regime.

## Methods

**Figure 2:**
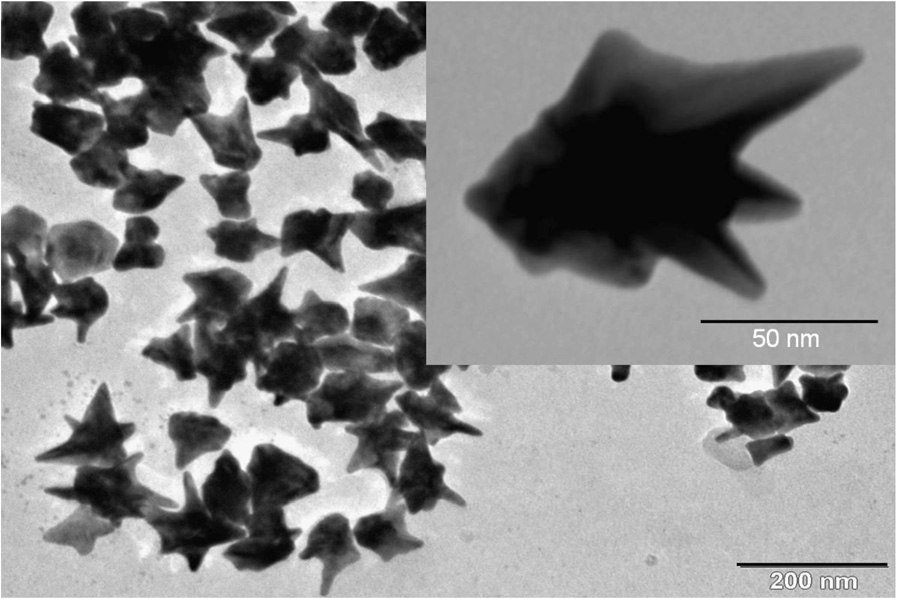
TEM image of star-shaped gold nanoparticles.

Gold nanostars were synthesized in an aqueous solution using cetyltrimethylammonium bromide (CTAB) as the capping and growth-regulating agent [17]. A transmission electron microscopy (TEM) image of nanostars (obtained using Philips CM20 TEM, Amsterdam, The Netherlands) is shown in Figure 2. TEM image of a single multispiked nanostar is shown as inset in Figure 2.

J-aggregates were formed from the following two dyes: JC1 (5,5^′^,6,6^′^-tetrachloro-1,1^′^,3,3^′^-tetraethyl-imidacarbocyanine iodide) and S2165 2-[3-[1,1-dimethyl-3-(4-sulfobutyl)-1,3-dihydro-benzo[e]indol-2-ylidene]-propenyl]-1,1-dimethyl-3-(4-sulfobutyl)-1*H*-benzo[e]indolium hydroxide. J-aggregates of the JC1 dye form spontaneously upon dissolution of this dye in deionized water at pH7, while the formation of J-aggregates of S2165 required the addition of polyethyleneimine (PEI). The reason why we choose these particular dyes was that upon aggregation they develop very narrow absorption bands (J-bands) both located very close to the maximum of nanostar absorption which favors the regime of strong plasmon-exciton coupling in hybrid systems.

**Figure 3:**
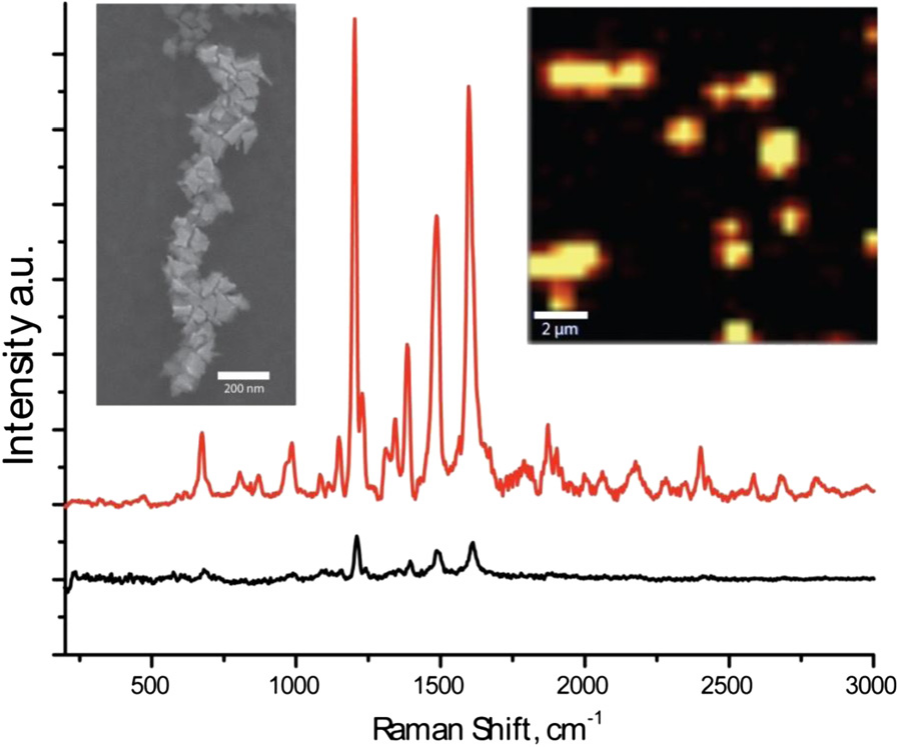
**Surface-enhanced Raman spectra, scanning electron microscopy image, and Raman micromapping.** Surface-enhanced Raman spectrum (red curve) of the hybrid nanostructure of gold nanostars and the J-aggregates of JC1 dye as compared to the Raman spectrum of J-aggregates of JC1 only (black curve). Scanning electron microscopy image and Raman micromapping of tightly bound agglomerates of gold nanostars and J-aggregates of JC1 dye are given in the left and the right insets, respectively.

Hybrid structures of gold nanostars and the J-aggregates of the JC1 dye were produced by the addition of the concentrated ethanol solution of the dye to an aqueous solution of gold nanostars in the presence of ammonia at pH8. Interactions between nanostars and JC1 molecules of J-aggregates resulted in the formation of chain-like tightly bound agglomerates of gold nanostars interconnected by an organic matter, with a typical appearance exemplified in the scanning electron microscopy image (obtained using an environmental scanning electron microscope Quanta 250 FEG, FEI, Hillsboro, OR, USA) in Figure 3. These agglomerates were separated from the excess of dye molecules or J-aggregates not bound to gold nanostars by centrifugation at 3,800 rpm for 2 min and redispersed in aqueous solution. CTAB, which was used in the synthesis of nanostars, is not only the shape-directing agent for anisotropic growth but also the stabilizer [17] which provides a net positive surface charge to the nanoparticles, making them suitable for the formation of agglomerates with oppositely charged species like J-aggregates due to electrostatic interactions [22-24]. In our case, these interactions favored the formation of chain-like organic/inorganic structures (Figure 3).

The formation of the hybrid structures of two constituent compounds has been further confirmed by surface-enhanced Raman scattering (SERS) measurements using a confocal Raman microscopy setup (Alpha300, 600 mm^−1^ grating, 3 cm^−1^ spectral resolution, continuous wave laser excitation at 532 nm, WITec, Ulm, Germany), as the hot spots provided by sharp tips of agglomerated Au nanostars are expected to enhance Raman scattering response of the attached organic compounds [18]. Indeed, the SERS spectrum of the hybrid nanostructures of gold nanostars and the JC1 J-aggregates (red curve in Figure 3) shows identical but by more than an order of magnitude enhanced features as compared to the conventional Raman spectrum of J-aggregates (black curve in Figure 3). Raman micromapping of hybrid gold nanostars/J-aggregate (JC1) complexes dispersed over a glass slide (Figure 3, right inset) directly demonstrates the strong enhancement of the Raman signal at the location of agglomerates.

## Results and discussion

**Figure 4:**
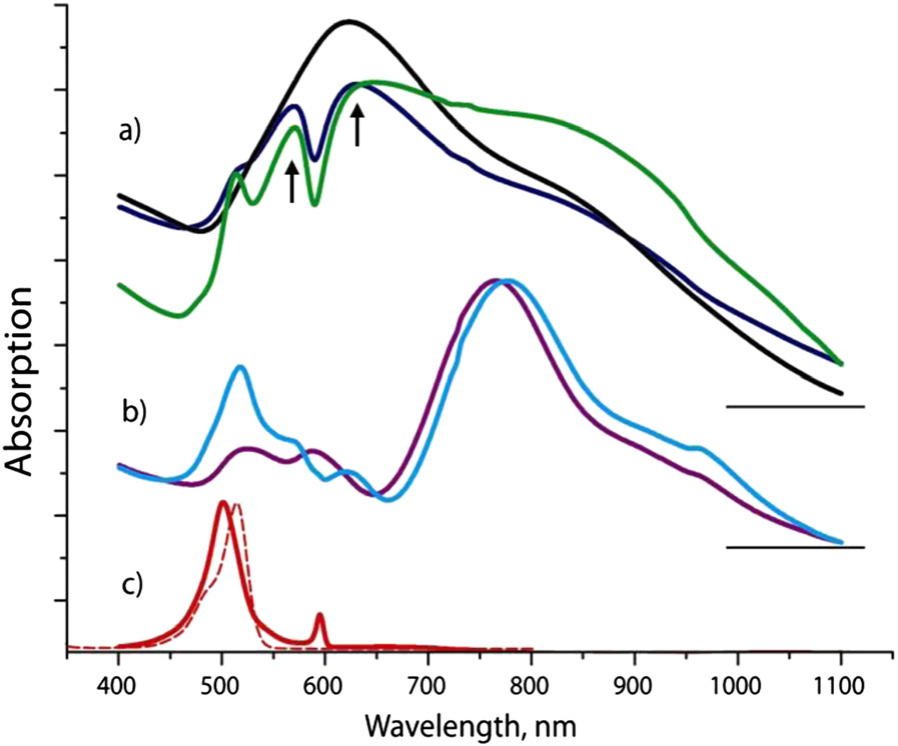
**Absorption spectra of the aqueous solutions. **(**a**) Gold nanostars (black) and their hybrid structures with J-aggregates of JC1 dye without (blue) and with PEI (green); (**b**) gold nanorods (violet) and their hybrid structure with J-aggregates of JC1 dye (cyan); (**c**) pristine J-aggregates of JC1 dye (red, solid line) along with the spectra of the solution of JC1 dye in methanol (red, dashed line). Horizontal lines indicate zero levels for each group of the absorption spectra.

The absorption spectrum of Au nanostars exhibits a broad, intense band centered at 623 nm, along with a less intense shoulder at 827 nm (Figure 4a, black curve). J-aggregates of JC1 show a narrow absorption band (J-band) at 595 nm with a full width at half maximum of 7 nm, alongside with a broader absorption band, positioned at the lower wavelength side from the J-band (at 500 nm) which we assign to the absorption of JC1 monomers (Figure 4c) [25]. JC1 dye has extremely poor water solubility, which favors the formation of J-aggregates even at 0.1 μM concentration. For this reason, the peak associated with J-aggregates is always present in the spectra of aqueous solution of JC1, which makes it difficult to measure the absorption spectrum of the dye monomers alone [25]. To ensure that the 500-nm peak assignment to monomer absorption is consistent, we have measured the spectrum of JC1 dye dissolved in methanol where (due to high solubility of the dye) its aggregation is inhibited and only the absorption band of dye monomers can be detected (peak at 517 nm in Figure 4c, dashed line). Taking into account small bathochromic shift caused by solvatochromism [26], this spectrum confirms the 500-nm band assignment.

In the hybrid structure of both nanostars and J-aggregates, the pronounced dip at 590 nm (which corresponds to the absorption wavelength of the J-aggregates) appears as a result of strong coupling of the excited states of J-aggregates and plasmon modes of the nanostars (Figure 4a, blue curve). The wavelength separation between the two peaks in this spectrum (indicated by arrows in Figure 4) is 61 nm, giving the value of Rabi splitting of 213 meV. This value depends on the total absorbance or, in other words, on the concentration of J-aggregates [27], which, for cyanine dye molecules used in this work, can be influenced by the addition of charged polyelectrolytes [28]. This is demonstrated in Figure 4a (green curve), where positively charged polyelectrolyte PEI has been added to gold nanostars and to the JC1 molecules. As a result, Rabi splitting energy increased to 260 meV, which is 13% of the total transition energy (which corresponds to spectral position of the dip), indicating the strong coupling regime between the plasmons and the J-aggregate excitons.

To demonstrate the advantage of using Au nanostars for the strong coupling with J-aggregates, it would be instructive to compare the values of the achieved Rabi splitting with that of a hybrid system consisting of J-aggregates and gold nanorods [29] of similar volume as nanostars. Based on the TEM image (Figure 2), the effective volume of nanostars was estimated approximating their inner core part by a sphere to which the spikes are attached.

The absorption spectrum of Au nanorods used here (Figure 4b, violet curve) exhibits two main resonances: the red-shifted peak at 766 nm corresponds to the longitudinal surface plasmon resonance, whereas the spectral position of the two other bands spanning over the region between 450 and 650 nm is consistent with the wavelengths of the transverse plasmon modes. The absorption band of J-aggregates of JC1 dye (Figure 4c) falls within the spectral region of the blue-shifted band of the nanorods. In the hybrid system of Au nanorods and J-aggregates, which was fabricated in a similar fashion as that of the gold nanostars, a dip at 595 nm (Figure 4b, cyan curve) with Rabi splitting of 185 meV is observed, which is a much smaller value than that demonstrated above for the nanostar-based hybrid system.

**Figure 5:**
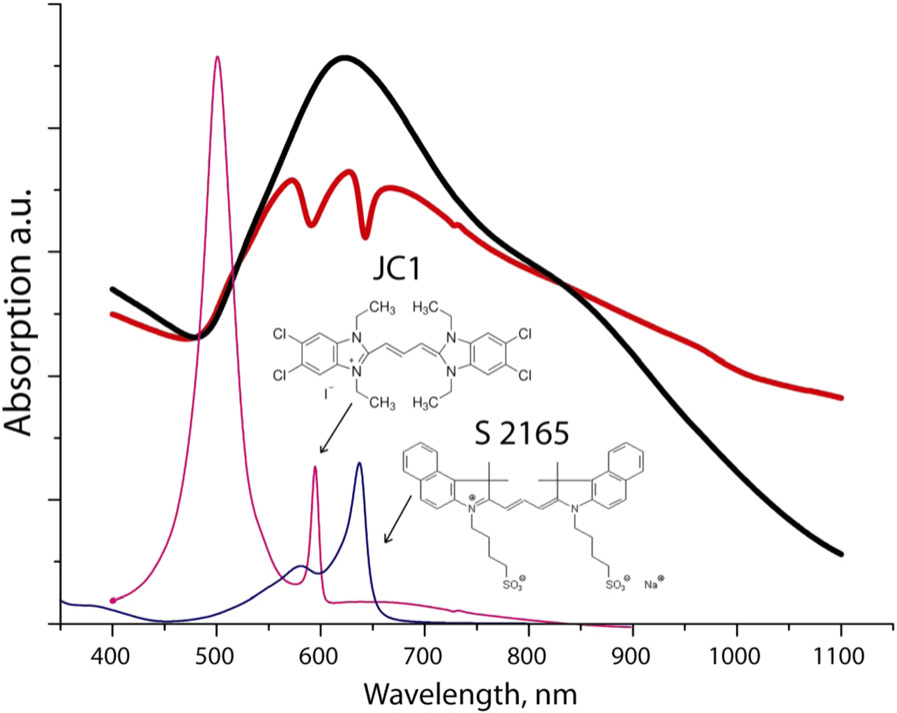
**Absorption spectra of gold nanostars, pristine J-aggregates of JC1 and S2165, and their hybrid structure.** Absorption spectra of gold nanostars (black curve) and their hybrid structure with J-aggregates of both JC1 and S2165 dyes (red curve). Absorption spectra of pristine J-aggregates of JC1 and S2165 dyes are shown in magenta and blue, respectively, together with their chemical structures.

Large number of localized plasmon modes in Au nanostars available for coherent coupling with integrated emitters provides the possibility to observe multiple Rabi splitting for the hybrid system where two (or more) different J-aggregate emitters are strongly coupled to gold nanostars. To demonstrate this possibility, we developed a more complex hybrid system integrating nanostars with J-aggregates of not only JC1 but also S2165 dye, whose absorption band is centered at 637 nm, and thus, more than 30 nm red-shifted with respect to the absorption band of JC1 J-aggregates (Figure 5). J-bands of both dyes still fall perfectly within the region of the main feature in the nanostar absorption spectrum. This matching provides a perfect condition for strong coupling. It is well known that the presence of charged polyelectrolytes enhances the tendency of cyanine dyes to form J-aggregates [28,30,31]. Moreover, as demonstrated above (Figure 4), the value of the Rabi splitting and therefore the strength of exciton-plasmon coupling can be increased by raising the concentration of J-aggregates, which, in turn, can be controlled by an addition of charged polyelectrolytes. For these reasons, the PEI polyelectrolyte has been used to induce the formation of J-aggregates of both dyes bound to gold nanostars. The absorption spectrum of the resulting complex hybrid system shows two pronounced dips at 590 and 642 nm (Figure 5, red curve), which correspond to the maximum absorption wavelengths of the J-aggregates of JC1 and S2165, respectively. Thus far, the double Rabi splitting was observed with the energies of 187 and 119 meV.

It is well known that in the strong coupling regime, the spectral lineshapes of the hybrid system can be interpreted interchangeably as a result of the plasmon-exciton hybridization (leading to the formation of two distinct mixed states (Rabi effect)) and also by the interference of different excitation pathways (Fano interference) [32]. In the last case, one of the paths is a discreet excitonic state and the other is a quasi-continuum plasmonic state (Figure 1). Depending on whether or not the plasmonic and excitonic resonances are exactly matching, the profile of Fano resonances goes from a symmetric dip to an asymmetric lineshape, respectively [33]. In line with this, the observed asymmetric profiles of both dips in Figure 5 can be interpreted as results of slight mismatch between main resonance in the spectrum of the nanostars and spectral positions of J-aggregate excitonic transitions.

The observed lineshape can be theoretically reproduced using the model of a hybrid nanostructure consisting of a gold nanostar core surrounded by two layers of different J-aggregates [10]. Because direct modeling of nanostar shape is very challenging, we used a more simple approach approximating their shape as an ellipsoid with three different radii and tried to match the experimental plasmon spectra of the nanostars. The dielectric constant of gold was described by the Drude model:

(1) $$\varepsilon_{\mathrm{Au}}\left(\omega \right)=\varepsilon_{\infty }-\frac{\omega_{p}^{2}}{\omega^{2}+i\gamma_{\infty }\omega },$$

where *ω* is the frequency of the incident field, *ω*_p_ is the bulk plasmon frequency, *γ*_*∞*_ is the collision rate of electrons in Au, and *ε*_∞_ is the high-frequency component of the Au dielectric function. The dielectric constant of J-aggregates covering Au nanostars was modeled by a Lorentzian lineshape:

(2) $$\varepsilon_{\mathrm{Jn}}\left(\omega \right)=\varepsilon_{\mathrm{\infty jn}}-f_{n}\frac{\omega_{0n}^{2}}{\omega^{2}-\omega_{0n}^{2}+i\gamma_{n}\omega },$$

where *f*_*n*_ is the reduced oscillator strength, *γ*_*n*_ is the line width, *ω*_0*n*_ is the transition frequency, and *ε*_∞*jn*_ is the high-frequency component of dielectric function of the first (*n* = 1) and second (*n* = 2) types of J-aggregates.

**Figure 6:**
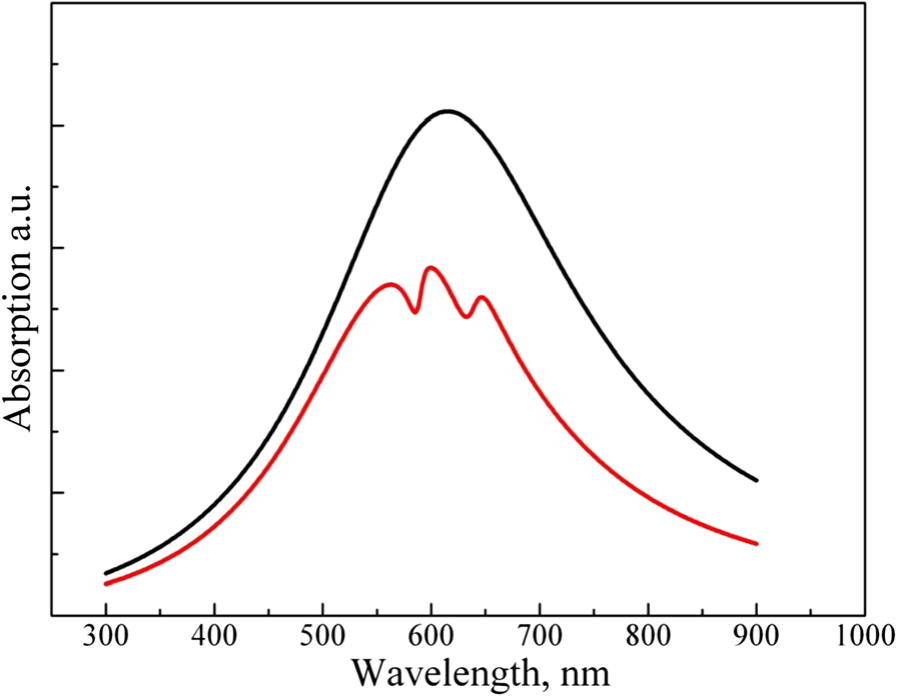
**Theoretical extinction spectra of gold nanostars (black) and their hybrid structure with J-aggregates (red curve).** The hybrid nanostructure has excitonic transition energies similar to those of JC1 and S2165 dyes.

The results from the model simulations (Figure 6) corroborated the experimental findings. As the positions of the excitonic resonances are shifted either to the red or to the blue with respect to the nanostar absorption maximum, distinctive asymmetric profiles can be seen in the spectrum of hybrid system.

## Conclusions

In conclusion, we introduced hybrid structures consisting of Au nanostars and J-aggregates of the cyanine dyes, where the coherent coupling between the localized plasmons of the metal component and the excitons of the J-aggregates reveals itself in Rabi splitting with the energy up to 260 meV. Owing to the remarkably broad features in the absorption spectra of gold nanostars, we were able to realize double Rabi splitting through their surface plasmon coupling to the excitons of two different dyes. This experimental finding paves the way towards the development on advanced hybrid systems and further investigations of the interaction between multiple emitters mediated by localized plasmons of different metallic nanostructures in the quantum electrodynamics regime. Alongside with the other multicomponent hybrid plexcitonic structures [32,34], hybrid systems realized and studied here offer a platform for the practical development of nanoscale optoelectronic and quantum information devices.

## Competing interests

The authors declare that they have no competing interests.

## Authors’ contributions

AS and DS carried out the synthesis, the assembly of hybrid structures, and the characterization experiments. DM designed and performed the experiments on optical properties of the samples and drafted the manuscript. AR and YR supervised the work and finalized the manuscript. All authors read and approved the final manuscript.
